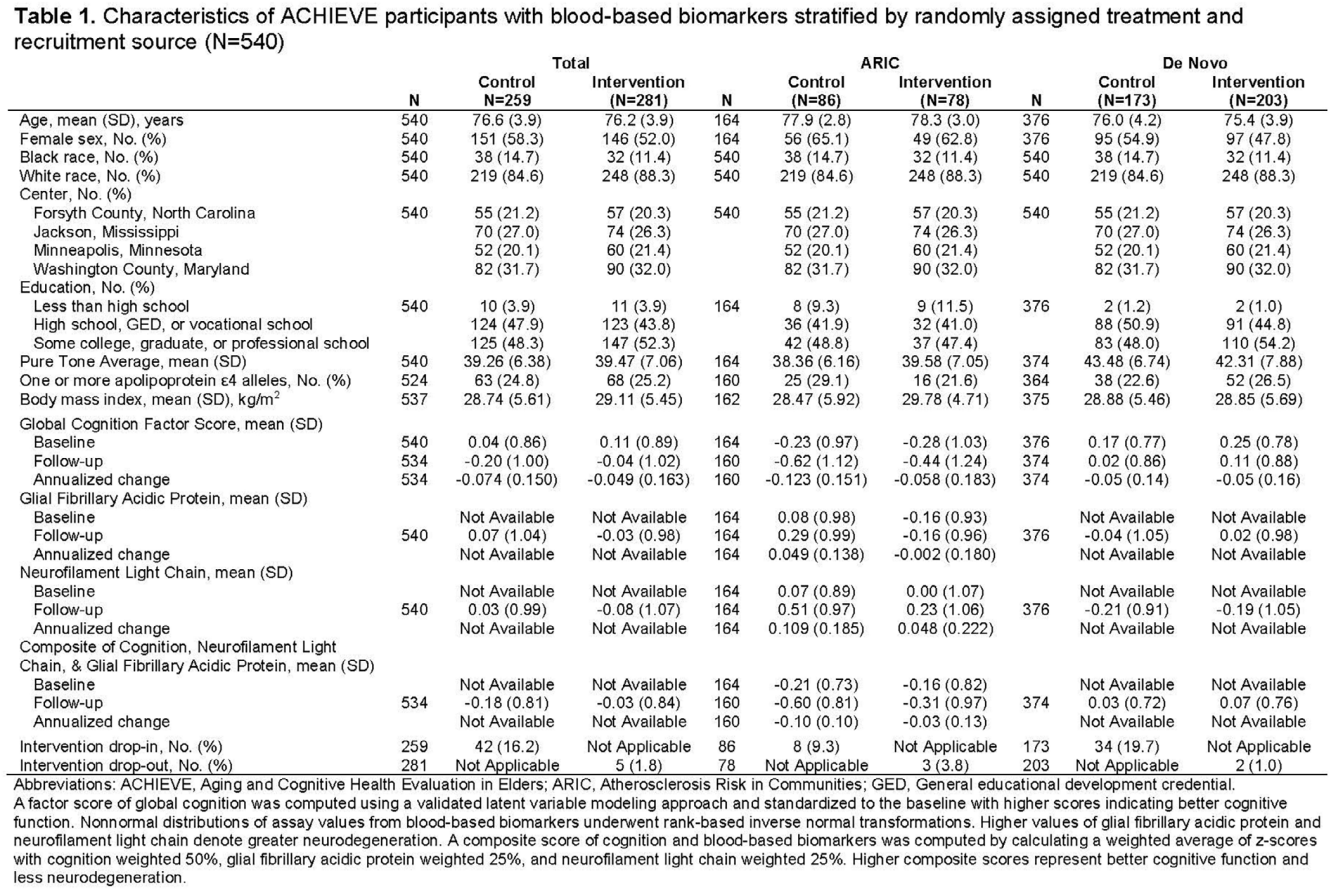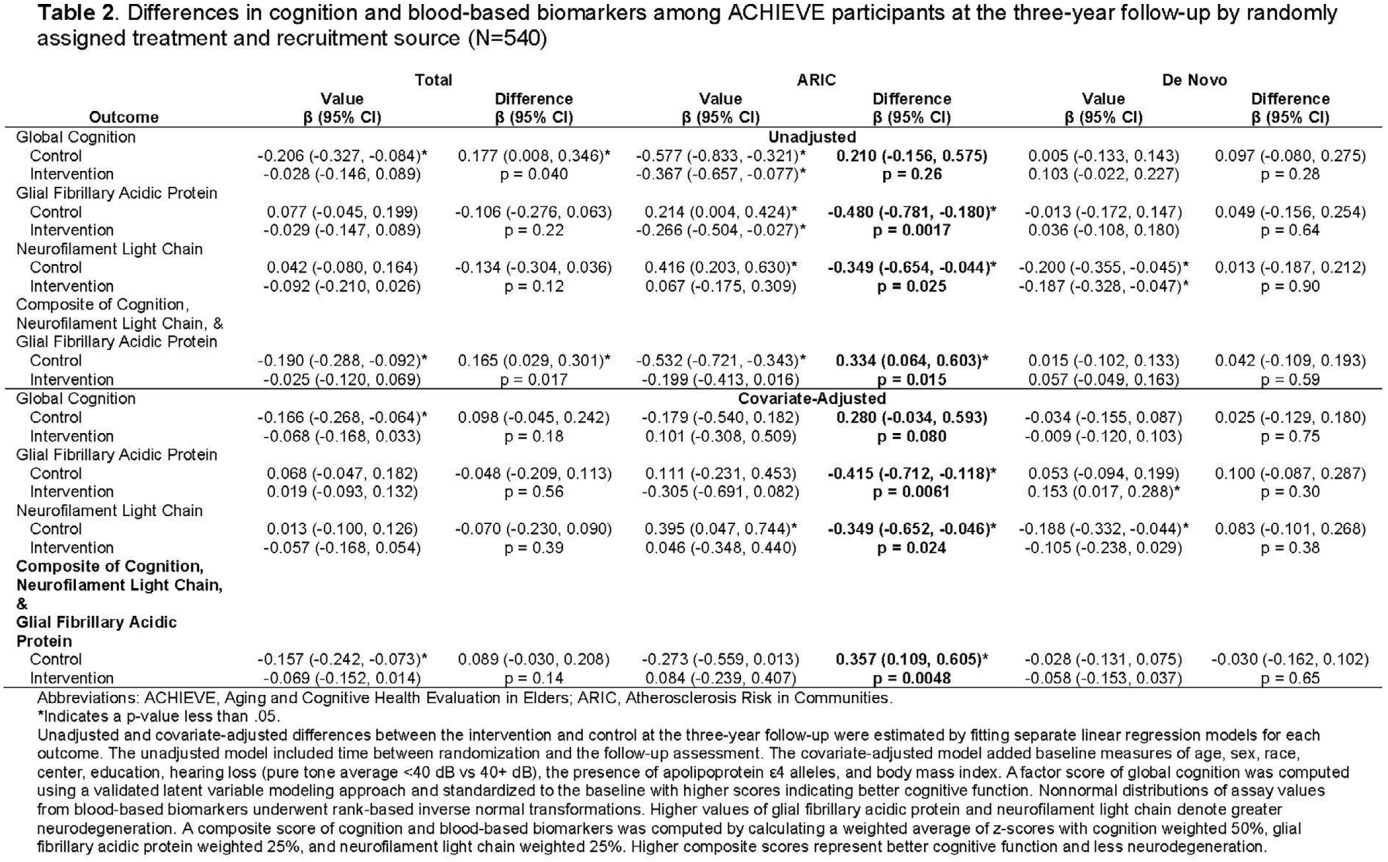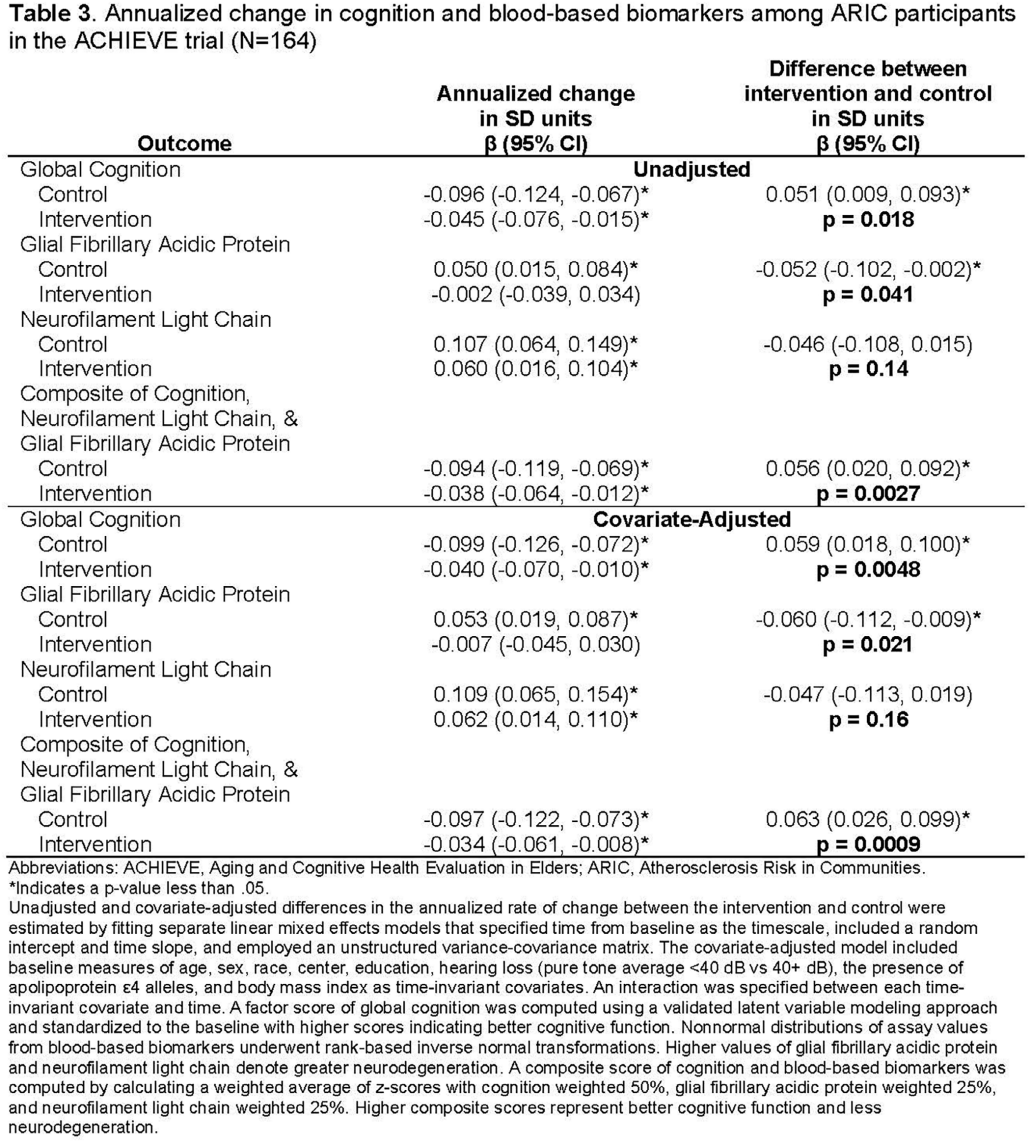# Effects on blood neurodegeneration biomarkers of a randomized hearing rehabilitation intervention: ACHIEVE Clinical Trial update

**DOI:** 10.1002/alz70860_107403

**Published:** 2025-12-23

**Authors:** Nicholas S Reed, James Russell Pike, Bharat Thyagarajan, Jennifer A. Deal, Michelle L Arnold, Theresa Chisolm, David Couper, Rebecca F. Gottesman, Timothy M. Hughes, David S. Knopman, Thomas H. Mosley, Priya Palta, James S Pankow, Victoria A Sanchez, Elizabeth Selvin, Kevin J. Sullivan, Lynne E Wagenknecht, Frank R Lin, Josef Coresh

**Affiliations:** ^1^ New York University, New York, NY, USA; ^2^ Departments of Population Health and Medicine, New York University Grossman School of Medicine, New York, NY, USA; ^3^ University of Minnesota, Minneapolis, MN, USA; ^4^ Johns Hopkins Bloomberg School of Public Health, Baltimore, MD, USA; ^5^ University of South Florida, Sarasota, FL, USA; ^6^ University of South Florida, Tampa, FL, USA; ^7^ University of North Carolina, Chapel Hill, NC, USA; ^8^ National Institute of Neurological Disorders and Stroke, Intermural Research Program, National Institutes of Health, Bethesda, MD, USA; ^9^ Wake Forest University School of Medicine, Winston‐Salem, NC, USA; ^10^ Mayo Clinic, Rochester, MN, USA; ^11^ University of Mississippi Medical Center, The MIND Center, Jackson, MS, USA; ^12^ University of North Carolina at Chapel Hill, Chapel Hill, NC, USA; ^13^ Johns Hopkins University Bloomberg School of Public Health, Baltimore, MD, USA

## Abstract

**Background:**

The Aging and Cognitive Health Evaluation in Elders (ACHIEVE) randomized trial (*n* = 977;ClinicalTrials.gov:NCT03243422) demonstrated that hearing intervention slowed 3‐year cognitive decline by 48% among a subgroup of participants. To further investigate differences by treatment with respect to cognitive benefit, we tested the hypothesis that hearing intervention is associated with improved neurodegeneration blood biomarkers 3‐years post‐randomization and assessed whether combining biomarkers and cognitive results improved power in the trial design.

**Method:**

The ACHIEVE study is a multicenter, parallel‐arm, randomized trial (hearing intervention vs health education control) on 3‐year cognitive decline among adults 70–84years with untreated hearing loss and without substantial cognitive impairment. Participants were recruited from two populations: (1) a long‐standing cardiovascular health observational study (Atherosclerosis Risk in Communities [ARIC]), and (2) de novo community volunteers. Plasma was collected in year 3 in a subsample (*n* = 540) while ARIC participants (*n* = 164) also had baseline plasma. Glial fibrillary acidic protein (GFAP) and neurofilament light (NfL) neurodegenerative biomarkers were derived using the Alamar CNS protein panel. Regression models estimated the association of treatment with 3‐year biomarkers and global cognitive differences in the combined sample and by recruitment source and annualized change from baseline in the ARIC sample. We included a simple composite score of the standardized outcomes (Table 2).

**Result:**

Among the subsample, baseline characteristics were balanced by treatment. There were no significant differences on 3‐year neurodegeneration biomarker levels by treatment in the combined (Table 1) or de novo groups (Table 2). Among ARIC participants, intervention resulted in lower 3‐year GFAP (mean:‐0.415;95%CI:‐0.712,‐0.118) and NfL (mean:‐0.349;95%CI:‐0.652,‐0.046) (Table 2). Annualized change from baseline analyses among ARIC participants (Table 3) revealed intervention was associated with a slower rate of cognitive decline (difference‐in‐means:0.059;95%CI:0.018,0.100) and a slower rate of increase in GFAP (difference‐in‐means: ‐0.060;95%CI:‐0.112,‐0.009). Differences in estimates suggest combined score, relative to cognition alone, may increase power in clinical trials that show a positive effect (ARIC subgroup: *p* = 0.02vs. 0.08[Table 2] and *p* = 0.0009vs.0.048[Table 3]).

**Conclusion:**

Hearing intervention was associated with positive 3‐year effects on neurodegeneration blood biomarkers in ARIC participants which parallels cognitive decline results. Results provides objective evidence of brain changes following hearing intervention and potential signal of a more powerful combined outcome approach for future brain health clinical trials.